# Spatial distribution of errors associated with multistatic meteor radar

**DOI:** 10.1186/s40623-018-0860-2

**Published:** 2018-06-05

**Authors:** W. K. Hocking

**Affiliations:** Department of Physics and Astronomy, University of Western Ontario, 1151 Richmond St. North, London, ON N6A 3K7 Canada

**Keywords:** Radar, Meteor, Errors, Multistatic, Specular, Reflection, Scatter

## Abstract

With the recent increase in numbers of small and versatile low-power meteor radars, the opportunity exists to benefit from simultaneous application of multiple systems spaced by only a few hundred km and less. Transmissions from one site can be recorded at adjacent receiving sites using various degrees of forward scatter, potentially allowing atmospheric conditions in the mesopause regions between stations to be diagnosed. This can allow a better spatial overview of the atmospheric conditions at any time. Such studies have been carried out using a small version of such so-called multistatic meteor radars, e.g. Chau et al. (Radio Sci 52:811–828, 2017, 10.1002/2016rs006225). These authors were able to also make measurements of vorticity and divergence. However, measurement uncertainties arise which need to be considered in any application of such techniques. Some errors are so severe that they prohibit useful application of the technique in certain locations, particularly for zones at the midpoints of the radars sites. In this paper, software is developed to allow these errors to be determined, and examples of typical errors involved are discussed. The software should be of value to others who wish to optimize their own MMR systems.
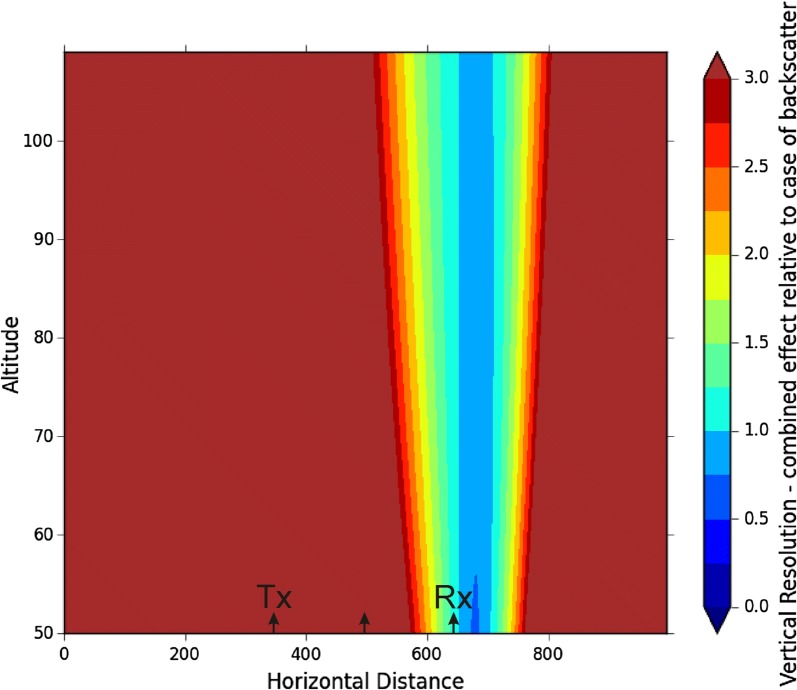

## Introduction

Monostatic interferometric meteor radars have been in existence for many decades, and their basic principles are well described in various texts (e.g. Hocking et al. 2001; 2016, Chapter 10). A substantial increase in meteor detection efficiency occurred in the late 1990s and early 2000s following the development of better techniques, spurring an increase in the deployment of such radars.

These radars rely on the fact that meteor trails are very effective radio-wave scatterers, and so radars of modest power (6–30 kW peak pulsed) can be used to detect them. As a result, there are many such radars worldwide. In some cases, such as in Europe and Scandinavia, there are a significant number of such radars within relatively close proximity (a few hundred to 1000 km or so separation) and so it has been proposed that these radars can be used in concert to study regions of the atmosphere between the radars, in addition to studies of the meteor field in the immediate proximity of each radar. Furthermore, remote receiving sites can be established relatively easily, so that one transmitter can be used by several receivers. GPS technology allows locking of the phases between the transmitter and receiver sites. Chau et al. (2017) has demonstrated the application of such techniques.

The radars usually transmit on a broad beam and receive on a cluster of receiver antennas—often 5 (e.g. Jones et al. 1998). Each receiver records separate signals, and by cross-correlating the complex signal measured on each receiver, interferometry may be used to determine echo location angles. Combined with range information, this allows complete location of the meteor trail. The signal may be further interrogated to determine decay times, atmospheric temperatures, atmospheric wind speed and other significant atmospheric parameters pertaining to the height region 75–100 km altitude.

However, care is always needed, as the signals are quite short-lived and can easily be confused with other impulsive signals like lightning and man-made ignition systems. Great care is needed to distinguish meteors from other short-lived phenomena, as discussed by Hocking et al. (2001).

Most interferometric meteors working at HF, MF and lower VHF frequencies (the most common frequencies for these types of radars) rely on so-called specular reflections from the meteor trail. For monostatic systems, this means that the trail must be orientated perpendicularly to the vector from the radar to the midpoint of the trail, so that the signal reflects back as if being bounced off a mirror. For bistatic systems, in which the transmitter and the receiver are not co-located, the angle of incidence of the wave from the transmitter relative to the meteor trail must equal the angle of reflection of the wave back towards the receiver location. We will develop our theory using the bistatic case: the monostatic case is simply a limiting case of the bistatic case, where the transmitter and receiver become coincident.

Resolution is always an issue, since many meteors are detected at significant angles from zenith (up to 60° from zenith), and resolution degrades at lower angles. With applications involving bistatic and multistatic systems, errors become even more of an issue. In this paper, we address these different errors and develop software to study them. The code is written in the *python* language and is presented in the Appendix. It may be used freely by the reader, provided it is properly acknowledged in any correspondence and publications. In the following discussions, we refer to the software frequently.

Our main interest here is in determination of errors involving (1) height resolution and (2) wind-velocity accuracy. These are discussed in the following "Height resolution" and "Velocity measurements" sections, followed by some discussion in the "Discussion" section and finally conclusions in the "Conclusions" section.

## Height resolution

Height resolution involves two separate aspects of the radar: (1) the pulse length and (2) the angular resolution. For meteors close to overhead of a monostatic radar, only (1) matters, but as soon as the meteors occur at significant off-zenith angles, angular effects contribute to the height resolution. For example, a meteor at 45° from overhead has vertical resolution of approximately √{((Δ*z*/√2)^2^ + (*r* Δ*θ*/√2)^2^}, Δ*z* being the pulse resolution and Δ*θ* being the angular resolution. If the angular resolution Δ*θ* is ~ 1°–2° (as is typical in many such systems), then the contribution of the angular component for a 1° angular resolution is of the order of 1.5 km. Meteor radars commonly use a pulse length of a similar value—typically 2 km—since there is no real improvement in resolution by using shorter pulses. (The angular effect often dominates at angles where meteors are most easily detected.) Wider pulses also require a narrower frequency allocation band and allow the use of narrower-band receiver filters (hence reducing noise and interference).

**Fig. 1:**
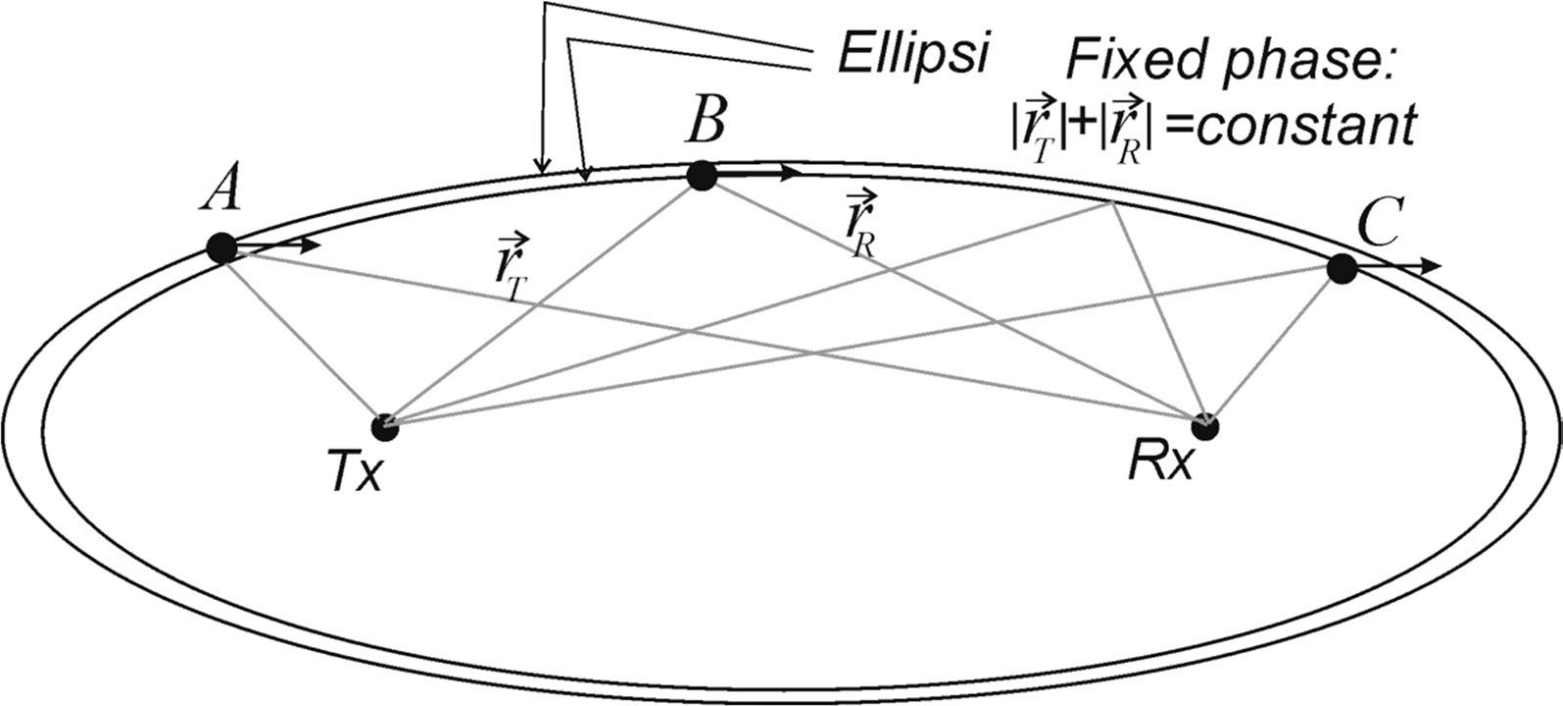
Sketch showing the nature of scattering in bistatic mode, showing a vertical cut through two surfaces of constant “range” delay

For a monostatic radar, the delay time between transmission of the pulse by the transmitter and reception by the co-located receiver is used to determine the range of the target through the relation *r *=* c*Δ*t*/2. Often receiver systems are even calibrated in terms of “range” by using this formula. For bistatic systems, the situation is a little more complex. Figure 1 shows the delay time associated with various paths. Any signal that moves from the transmitter to a target and back to the receiver has the same time delay as long as the target is located on a common ellipse with foci at the transmitter and receiver. In Fig. 1, two closely located ellipses with the same foci are shown. If the targets move, then they may follow the arrows indicated at scatterers A, B and C. For meteor targets, each of the meteor trails must be aligned with an orientation perpendicular to a line which bisects the position vectors from the transmitter to the target and the target to the receiver. (The case for a non-specular scatterer is discussed in Hocking et al. 2016, Fig. 3.20.)

**Fig. 2:**
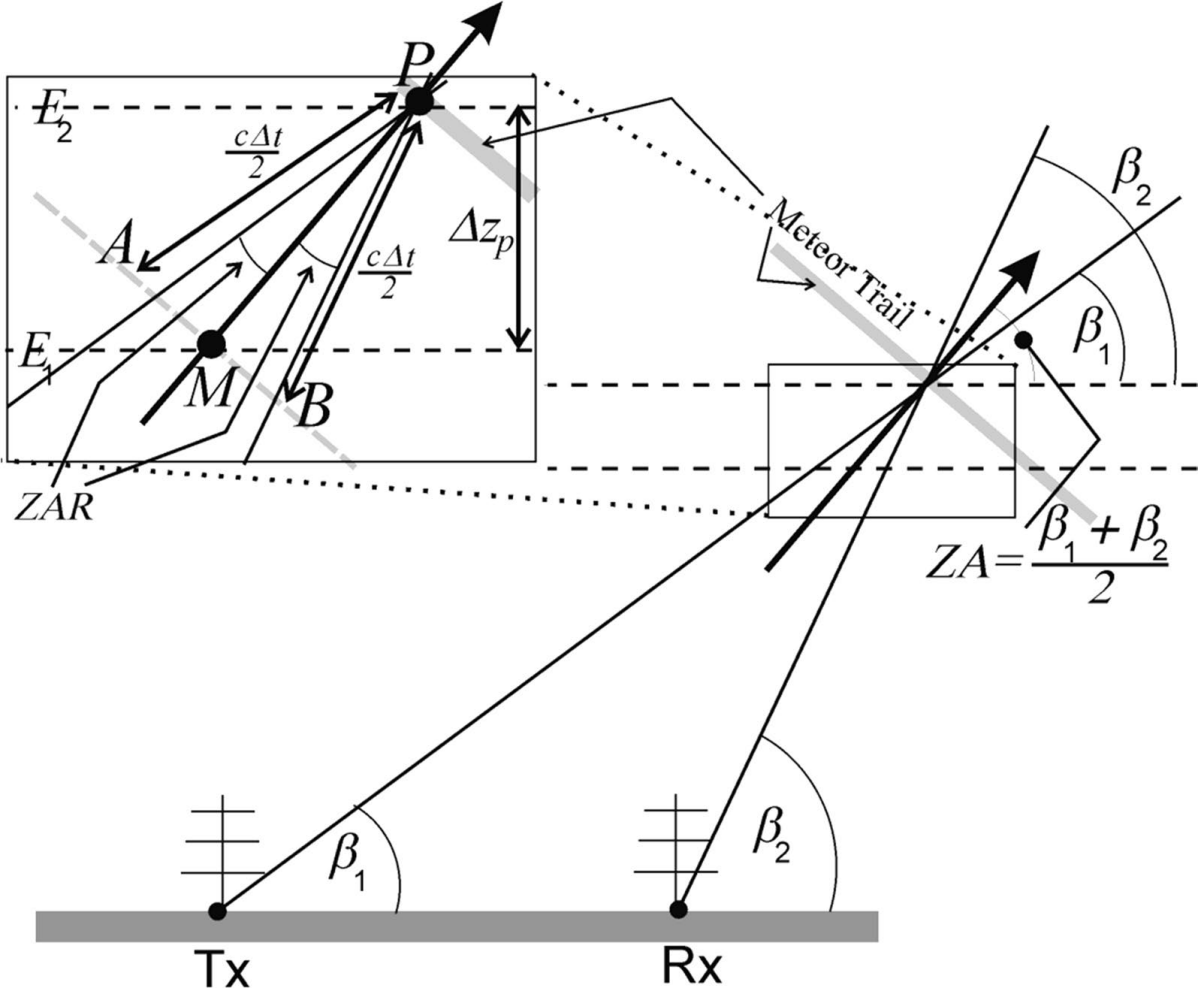
Orientation of a meteor trail, and path lines of ray vectors, for a general meteor. The radio pulse travels from the transmitter to the meteor trail and back to the receiver on the left. Note that only one receiver is drawn—normally there are more (typically 5) at separations of only one or two wavelengths from each other

In Fig. 2, scatterer A crosses from the outer ellipse to the inner one, and so sees a decrease in path length as time progresses. The time-rate-of-change of path length appears as a Doppler shift in the received signal and may be used to calculate a component of the wind strength. Scatterer B moves almost parallel to the inner ellipse and so shows no rate-of-change of path length, and so appears stationary to the receiver (no Doppler shift). No useful horizontal velocity component can be measured here, although the radar will be quite sensitive to vertical movement. Scatterer C moves from the inner to the outer ellipse and so shows a change in path length which can be translated to velocity component. Velocity sensitivity will be discussed in the next section.

With regard to range resolution, turn to Fig. 2. We concentrate on the vertical resolution due to the pulse length.

### Pulse-length effect on Δ*z*

A meteor trail is shown orientated perpendicular to the thick solid arrow, where the arrow bisects the lines from the transmitter and the receiver. The elevation of the meteor trail from the perspectives of the transmitter and the receiver is *β*_1_ and *β*_2_, respectively. The thick arrow is at an elevation of *ZA *= (*β*_1_ + *β*_2_)/2 and so bisects the lines from the transmitter and the receiver (thereby ensuring that the meteor is specularly reflecting). Note that meteors at the positions A, B and C in Fig. 1 will all have different orientations in order to satisfy specular reflection. [It is conceivable that when multiple meteor transmitters and receivers are used, the locations of the transmitters and receivers could be arranged so that under some circumstances a single meteor might reflect signals along different ray paths, thereby enabling two measurements of the Doppler shift and hence determination of a full vector at one place. As a warning, this does not work. Even if two reflections occur from the same trail, it will generally be from different portions of the trail, and turbulence effects will result in the different portions moving at different speeds. Such a procedure can be used to estimate turbulent strengths, however (e.g. Roper 1966).]

Now turn to the inset of Fig. 2. The meteor trail passes through *P*. We consider the instant at which the centre of the transmitted pulse rests at *P*. The leading half of the pulse has already passed *P* and is now at *B*, where the distance *BP* is *c*Δ*t*/2. The trailing half of the pulse is in the region between A and P, which also has a length *c*Δ*t*/2. The distance along the direction *MP* is therefore *c*Δ*t*/2 cos (*ZAR*) where *ZAR* is the bisection angle indicated in the figure and so equals (*β*_2 _− *β*_1_)/2. The vertical resolution is then Δ*z* as shown in the figure, which is just *MP* sin(*ZA*). Then, the resolution due to the pulse length is

1 $$\Delta z_{\text{p}} = c\Delta t/2\cos \left( {ZAR} \right)\sin \left( {ZA} \right)$$

The notations *ZAR* and *ZA* match the coding in the Appendix. Note that for a scatterer like B in Fig. 1, the transverse separation between A and B is quite large and could be considered to some extent as a contribution to the angular resolution uncertainty in the case of volume scatter. However, because the meteor is a discrete target, there is no such contribution here.

If the reader runs the code in the Appendix, the first two graphs produced will show the variation of Δ*z*_p_ as a function of position of the meteor. At an altitude of 90 km, the resolution varies from *c*Δ*t*/2 at the overhead point to a small fraction of *c*Δ*t*/2 at low elevations. We will not plot the graph here in order to save space.

### Angular vertical resolution

**Fig. 3:**
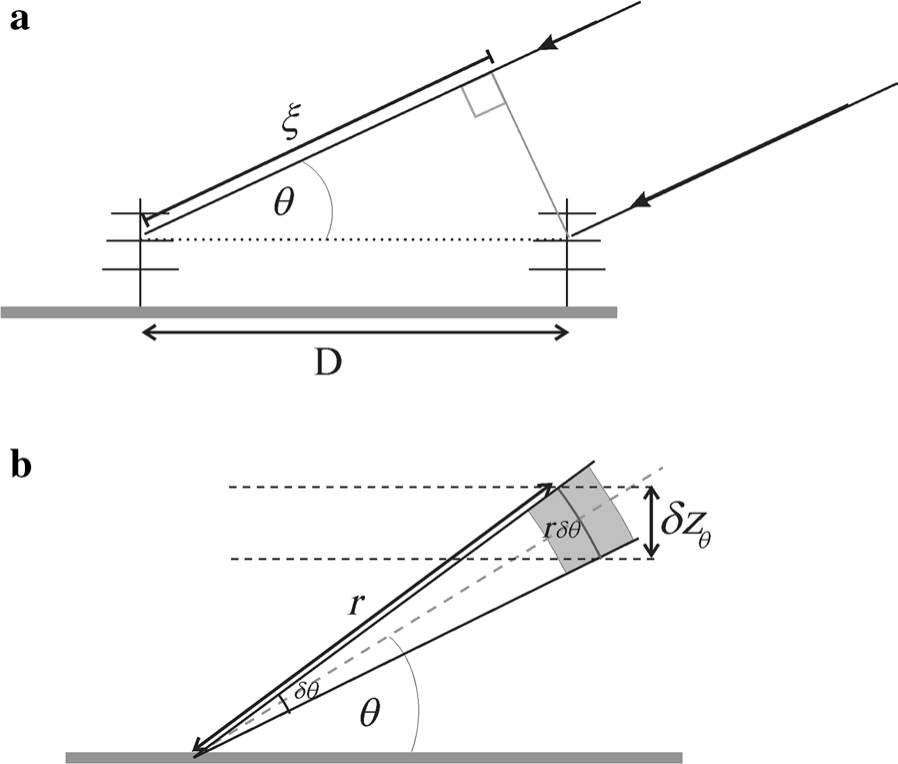
**a** A simple 2-antenna interferometer, for illustrative purposes. **b** Spatial resolution corresponding to an angular resolution *δθ*

Figure 3 shows two antennas of an interferometer. Radar signals enter from the right, as shown by the two arrows. We assume that the target is far enough away that the two rays can be considered as parallel. The two rays will be in phase along the perpendicular line, since they both were reflected coherently from the same target, and originated from the same pulse. The phase difference at the two receivers is therefore 2*πξ*/*λ*.

In application of interferometric techniques in 3D, there are at least 3 and generally 4 or 5 receivers. The receivers are placed at least one wavelength apart, to reduce antenna–antenna coupling, but this introduces redundancies into the possible directions. Therefore, there are usually more antennas than might be considered necessary, in order to resolve ambiguities. The SKiYMET system is a typical example (Hocking et al. 2001) and uses 5 antennas in the form of a cross (e.g. see Hocking et al. 1997, Fig. 1). The four outermost antennas are used to determine the possible meteor locations, but a multiplicity of possibilities exist due to angular ambiguities. Up to 6 and even 10 possible locations occur. Then, the fifth (central) antenna is used to determine which of the ambiguous positions is the correct one. Having found this correct position, a simulation is performed in real time in the radar controller to determine what the phase differences between all the antenna pairs should be, and this is compared with the actual phase differences. Invariably the differences are not zero. The phase differences can vary from the simulation by several tens of degrees. This is a result of noise, interference and imperfections in the assumption of specular reflection. The spread in phase errors limits the angular accuracy with which the target can be located.

With the SKiYMET system, this phase difference variation is generally limited to 35°; meteors with larger maximum phase errors are discarded. This value has been set empirically—large values allow acceptance of more meteors—even doubling the counts—but also allows many other false targets, and degrades the height resolution noticeably. Limits of less than 35° restrict the acceptable meteors too severely.

So assuming that each meteor recorded has this maximum phase error, we may place some limits on the angular resolution. This will be an upper limit, since often the maximum phase difference is less than our prescribed limit.

Then, from Fig. 3, the true phase delay can be written as Δ*ϕ *=* 2πξ/λ *=* 2π D*cos*θ/λ.* However, we seek the possible errors in Δ*ϕ*, which we will denote *δ(*Δ*ϕ*). Differentiation produces

2 $$\delta \left( {\Delta \phi } \right) = [( - 2\pi D\sin \theta )/\lambda ]\delta \theta .$$

Let the distance to the target meteor be *r*, where we consider this to be about the same for each antenna, since the target is over 90 km away and the antennas spacing is only a few metres. If the meteor is at height *z*, then sin*θ *=* z*/*r*, so (2) can be rewritten as

$$\delta \theta = - \frac{{\lambda \delta \left( {\Delta \phi } \right) r}}{2\pi D z}$$

From Fig. 2b, *δz*_*p*_ is clearly (*rδθ*) cos*θ,* so that

3 $$\delta z_{\theta } = - \frac{{\lambda \delta \left( {\Delta \phi } \right) r^2}}{2\pi D z}\cos \theta$$

In the program in the Appendix, *D/λ* is just the variable “*antrx*”, and we take *δ(*Δ*ϕ*) = 35° (converted to radians). Note that *r* is the distance from the centre of the receiver array and is unrelated to the transmitter position. This introduces asymmetries that will appear later. The variable *δz*_*θ*_ occurs in the Appendix as the variable “DTANG” and is then normalized relative to *c*Δ*t*/2.

### Total resolution

**Fig. 4:**
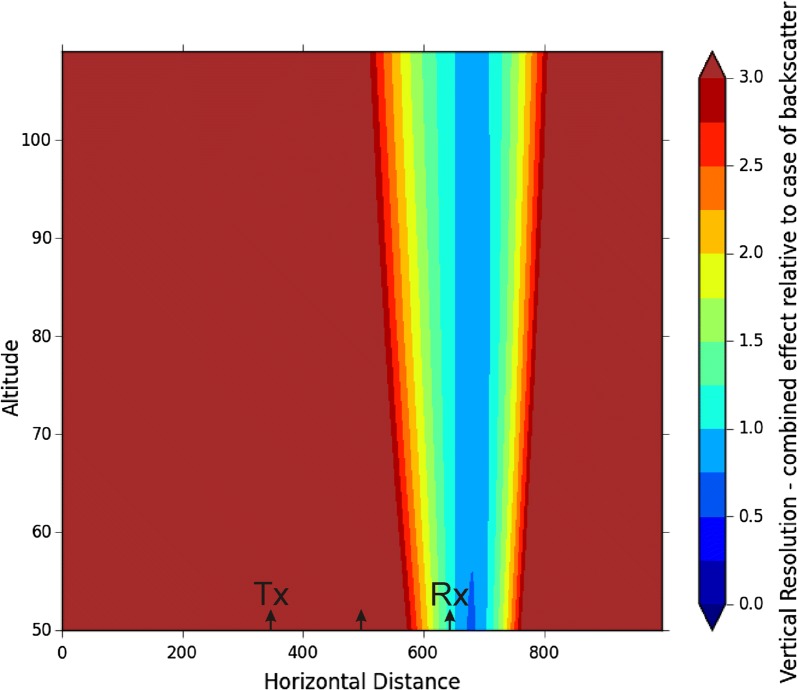
Vertical resolution of the system relative to the “vertical backscatter resolution”. Only the data between 75 and 110 km altitude are of interest here. See text for details

The program in the Appendix plots *δz*_*θ*_ as a function of position, but we will not show it here in order to save space. Rather, we combine the effects of Eqs. (1) and (3) by plotting the normalized total error, which is found by adding the squares of (1) and (3) (after each is normalized relative to *c*Δ*t*/2), and taking the square root. The result is shown in Fig. 4, for the case of a transmitter at *x* = 350 km and the receiver at *x* = 650 km. Only values up to 3 times *c*Δ*t*/2 are plotted—poorer resolutions are of no value to us and appear as a brown/dark-red colour. (At large distances, the angular effect of the system resolution clearly translates to very large vertical resolutions.)

Clearly only the region immediately above the receiver array produces data with suitable resolution. Meteors further away (including over the transmitter) offer little useful information.

The fact that the transmitter is 300 km from the receivers also obviously will result in significant loss of useful power before the radar signal reaches the meteors in the vicinity of the receiver, and in addition the transmitter pulses that reach these meteors will need to be transmitted at low elevations (in this case atan(300/90) = 73° from zenith). This will further reduce the signal arriving overhead because the signal will be transmitted at angles where the polar diagram of the transmitter antenna has weak values.

## Velocity measurements

Now we turn to the topic of measurements of the drift speed of the trail, which may be used to determine the wind velocities over the radar when combined with measurements with other radars. We will look at the Doppler shift of the reflected signal as it arrives at the receiver. For a monostatic system, this is referred to as the “radial velocity”; while the term is not really pertinent here, we will use it in a loose sense at times.

To do this, we return to Fig. 1. We will consider a generalized point representing any of the scatters A, B or C in the figure and calculate the distance from the transmitter to the scattering point and on to the receiver. Then, we will allow the scattering point to move horizontally a distance *vδt*, where *δt* is a small time interval (typically 0.1 s), and *v* is the velocity of interest. Then, the change in distance is divided by the time interval to give the rate of change of distance with time. This will result in a Doppler shift of the radiowaves, but because the meteor trail acts like a mirror, the measured Doppler shift will appear as twice the speed of the meteor. We therefore need to divide by 2 to get the true “radial” velocity.

**Fig. 5:**
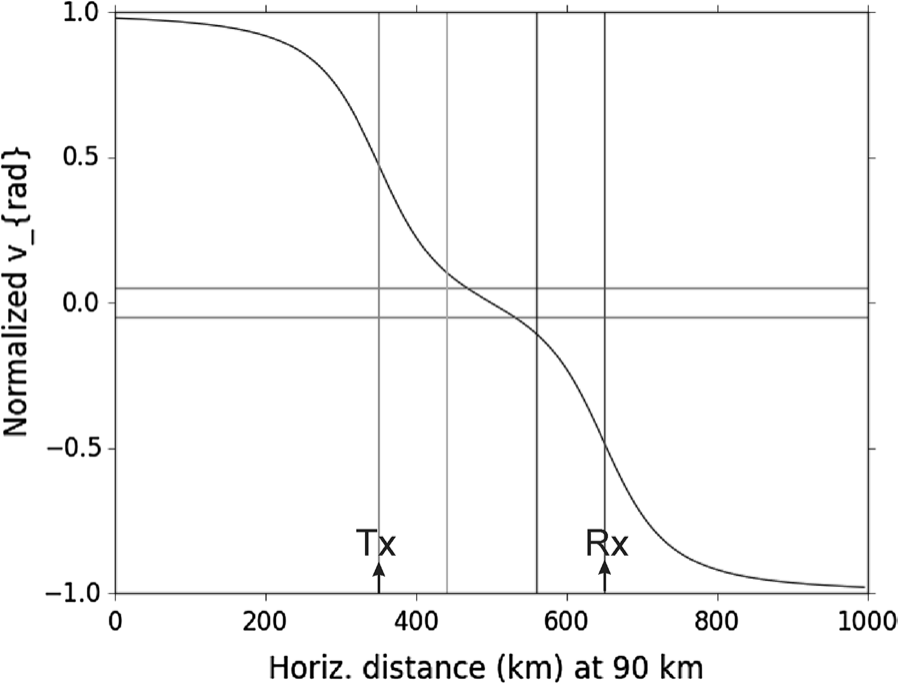
Doppler-shifted velocity components measured by the radar as a function of horizontal displacement at 90 km range, for a transmitter at *x *= 350 km and a receiver at *x *= 650 km. The speeds are normalized by division by the true horizontal velocity, so approach unity at the edges. The radar-measured parameter is referred to as *v*_rad_, by analogy with the backscatter case, but it is not truly a radial velocity. Lines are drawn horizontally near the zero-point on the abscissa—values within these lines will have measured speeds less than 0.05 of times the true horizontal speed, making inversion of the data difficult and potentially unreliable

The results at 90 km altitude for a transmitter–receiver of 300 km separation are seen in Fig. 5. Results are normalized relative to the true horizontal velocity. The transmitter was at *x *= 350 km and the receiver at *x* = 650 km. As seen, at low elevations (*x* = 0 and *x* = 1000) the measured velocity approaches that of the true horizontal velocity, confirming that the correct normalization has been used. Some extra lines have been added to the graph, as discussed in the figure caption. Data between *x *= 450 and *x *= 550 km give very small Doppler-shifted velocities and could have substantial errors in inversion. The Doppler-shifted values also change quite sharply as a function of distance beyond 550 km.

## Discussion

The results of Fig. 5 suggest that reliable velocities are likely to the right of *x* = 550 km. The results of Fig. 4 suggest that the resolution is unreliable at values of *x* to the left of 550 km and to the right of about 750 km. It would therefore appear that the bistatic radar is only really useful above the receiver system, between *x* = 550 and 750 km, i.e. within ± 100 km of the receiver system. Within this region, the ratio of the measured radar-determined component relative to the true speed changes rapidly, potentially introducing more errors.

It should also be noted that the calculations of Fig. 5 do not include height error considerations explicitly, so uncertainties in meteor trail location at low zeniths could add further errors.

In the "Pulse-length effect on Δ*z*" section, it was noted that the meteor trail is a specular reflector, while our height-error determinations were done on the basis of an assumed volume scatter. Because the meteor trail is discrete, it is possible to essentially deconvolve the pulse received at the receiver and determine the true trail range to better accuracy than the theory suggests, i.e. the position of the peak as a function of “range” will be a good representation of the true position. This could help reduce the height uncertainty but will, however, require that the signal is digitized at higher resolution than 2 km. However, at distances more than 100 km to the left or right of the receiver, the dominant cause of worsening resolution is the angular effect ("Angular vertical resolution" section), so the corrections for range achieved by deconvoution offer only limited opportunity for improvement.

Finally, we recall that the losses in effective power from the transmitter due to the long ranges involved and the low elevations required for the transmitter pulse passage will result in power losses in excess of 10 and up to 15 dB. Procedures might be developed to direct the transmitter signal more strongly at low elevations, but such considerations are beyond the scope of this paper.

## Conclusions

This paper sets limits on the capabilities of bistatic meteor radars and provides software that may be used to investigate range-height errors, angular errors and velocity inversion limitations under various circumstances. Preliminary results suggest that it is not possible to use meteors with confidence over some areas of the sky, while the region immediately above the receiver produces the most reliable data. However, software is provided to allow users to probe their own particular situations more carefully and perhaps fine-tune their systems for optimum performance.
